# Microfluidic-based systems for the management of diabetes

**DOI:** 10.1007/s13346-024-01569-y

**Published:** 2024-03-20

**Authors:** Shuyu Zhang, Anne E. Staples

**Affiliations:** 1 Virginia Tech-Wake Forest School of Biomedical Engineering and Sciences, Blacksburg, VA 24061 USA; 2 Department of Biomedical Engineering and Mechanics, Virginia Tech, Blacksburg, VA 24061 USA

**Keywords:** Microfluidics, Diabetes, Insulin, Drug delivery, Glucose monitoring, Glycated hemoglobin, Metformin, Microneedle array

## Abstract

Diabetes currently affects approximately 500 million people worldwide and is one of the most common causes of mortality in the United States. To diagnose and monitor diabetes, finger-prick blood glucose testing has long been used as the clinical gold standard. For diabetes treatment, insulin is typically delivered subcutaneously through cannula-based syringes, pens, or pumps in almost all type 1 diabetic (T1D) patients and some type 2 diabetic (T2D) patients. These painful, invasive approaches can cause non-adherence to glucose testing and insulin therapy. To address these problems, researchers have developed miniaturized blood glucose testing devices as well as microfluidic platforms for non-invasive glucose testing through other body fluids. In addition, glycated hemoglobin (HbA1c), insulin levels, and cellular biomechanics-related metrics have also been considered for microfluidic-based diabetes diagnosis. For the treatment of diabetes, insulin has been delivered transdermally through microdevices, mostly through microneedle array-based, minimally invasive injections. Researchers have also developed microfluidic platforms for oral, intraperitoneal, and inhalation-based delivery of insulin. For T2D patients, metformin, glucagon-like peptide 1 (GLP-1), and GLP-1 receptor agonists have also been delivered using microfluidic technologies. Thus far, clinical studies have been widely performed on microfluidic-based diabetes monitoring, especially glucose sensing, yet technologies for the delivery of insulin and other drugs to diabetic patients with microfluidics are still mostly in the preclinical stage. This article provides a concise review of the role of microfluidic devices in the diagnosis and monitoring of diabetes, as well as the delivery of pharmaceuticals to treat diabetes using microfluidic technologies in the recent literature.

**Graphical abstract:**

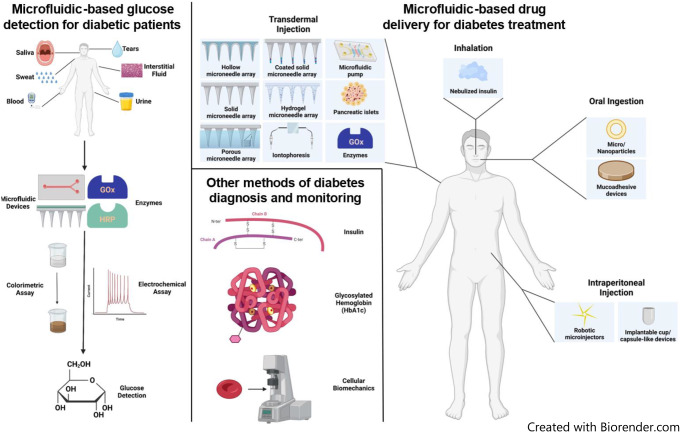

## Introduction

Diabetes is an extremely common condition that affects approximately 500 million people worldwide. This number is expected to grow to over 700 million by 2045 [1]. In the United States alone, approximately 37 million (11.3%) live with diabetes, and this statistic is expected to increase to 60.6 million in 2060 [2, 3]. The annual cost spent on diabetes in the United States was $327 billion in 2017, and diabetes is among the top five leading causes of death in the US [4–6]. According to Mobasseri et al. (2020), Type 1 diabetes (T1D), an autoimmune disease that causes a deficiency in insulin secretion, accounts for between 5% and 10% of all cases of diabetes. The rest of diabetic patients have type 2 diabetes (T2D), in which the body has an impaired ability to respond to insulin [7]. T1D patients typically experience more severe symptoms such as ketoacidosis, uncontrolled hyperglycemia, and hypoglycemia, as compared to T2D patients [8].

The diagnosis and monitoring of diabetes mainly relies on blood tests, most prominently finger-prick blood glucose tests [3, 9–16]. These blood tests are convenient, yet they pose limitations including pain, invasiveness, fear of needles, stress, potential infections, and non-healing of the penetrated area [3, 9, 11, 13–17]. These limitations could result in non-adherence to glucose monitoring, which in turn could cause complications including diabetic ketoacidosis, cardiovascular diseases, stroke, and blindness [10]. To eliminate these drawbacks, researchers have developed less invasive alternatives, which are already in clinical use. These alternatives include non-invasive blood glucose tests [17], as well as glucose tests based on other body fluids including interstitial fluid [18], saliva [9], sweat [19], tears [11], and urine [20]. Besides testing for glucose, researchers can also diagnose and manage diabetes based on the concentrations of glycated hemoglobin (HbA1c or simply A1c) and insulin in the blood [21–26], as well as red blood cell (RBC) and neutrophil mechanics and behavior [27–31].

Treatment of diabetes is centered around the delivery of insulin for all T1D patients because external insulin is necessary to maintain glycemic control and prevent ketoacidosis [8]. Approximately 20–30% of T2D patients are prescribed insulin, including 40% of T2D patients in the United States [32, 33]. T2D can also be treated with other peptides such as glucagon-like peptide 1 (GLP-1), as well as orally consumed small-molecule drugs such as metformin [34–39]. The majority of T1D patients and a small portion of T2D patients in the United States rely on battery-powered infusion pumps for insulin delivery, while the rest of insulin-dependent patients rely on cannula-driven insulin syringes or pens [8, 32]. All current methods of insulin delivery have associated side effects, the most obvious of which is painful cannula insertion [40–47]. In addition, embarrassment, interference with daily activity, and sometimes cost, are other limitations associated with these methods [48–53]. These limitations result in non-adherence to insulin therapy, which can cause hospitalizations and mortalities [48–51, 54, 55].

Microfluidic technologies may be key to alleviating some of the unpleasant side effects of insulin delivery that lead to non-adherence. A microfluidic device is defined as a system involving micrometer-scale channels and chambers and containing small volumes (microliters, nanoliters, or even smaller) of fluids [56–58]. Compared to conventional drug screening and delivery methods, advantages of microfluidic systems include compact size, precise dosage control, rapid and high-throughput analysis, reduced chemical waste, and reduced invasiveness [59]. In diabetes management, microfluidics has been used in preclinical and clinical studies in diagnosing and treating diabetes. In this article, we will review microfluidic technologies for diagnosing and monitoring diabetes, as well as microfluidic devices for delivering insulin and other pharmaceuticals for the treatment of diabetes.

## Use of microfluidic devices in diabetes diagnosis and monitoring

Microfluidic devices have been increasingly employed in diagnosing and monitoring diabetes in the last two decades, most commonly through testing of glucose levels in different bodily fluids. Alternatively, some researchers leveraged other techniques, such as the measurement of HbA1c and insulin in blood, and the examination of mechanical deformability of RBCs. A summary of these technologies is provided in Table 1 below.

**Table 1:** Summary of literature for microfluidic diagnosis and monitoring of diabetes [24, 27, 60–98]

Biomarker monitored	Body fluid used	Reference
Glucose	Blood/Plasma/Serum	[60–71]
	Interstitial fluid	[72–79]
	Saliva	[66, 68, 80–86]
	Sweat	[60, 83, 87, 88]
	Tears	[83, 89, 90]
	Urine	[66, 68, 91–93]
Glycated hemoglobin (HbA1c)	Blood	[94–96]
Insulin	Serum	[24, 97, 98]
Red blood cell deformability	Blood	[27]

### Devices for and methods of glucose detection

In miniaturized systems for glucose testing, microfluidic paper-based analytical devices (µPADs) have been the most commonly used due to their environmental friendliness, sustainability, biocompatibility, light weight, ease of transport and storage, as well as fast, easy, and inexpensive fabrication [60, 80, 81, 99]. These devices are typically fabricated with wax printing, which involves the pre-designing and patterning of molten wax or solid ink on choreographic paper, followed by the cooling of the paper to room temperature [99]. Other fabrication methods include photolithography [60], origami [61, 91], deposition with 3D pens [81], and CO2 laser cutting [89]. Besides paper-based devices, other device types include polydimethylsiloxane (PDMS) devices [72, 87], plexiglass chips [73], microfluidic thread-based electroanalytical devices (µTEDs) [66, 90], and porous microneedle arrays [74, 75].

**Fig. 1:**
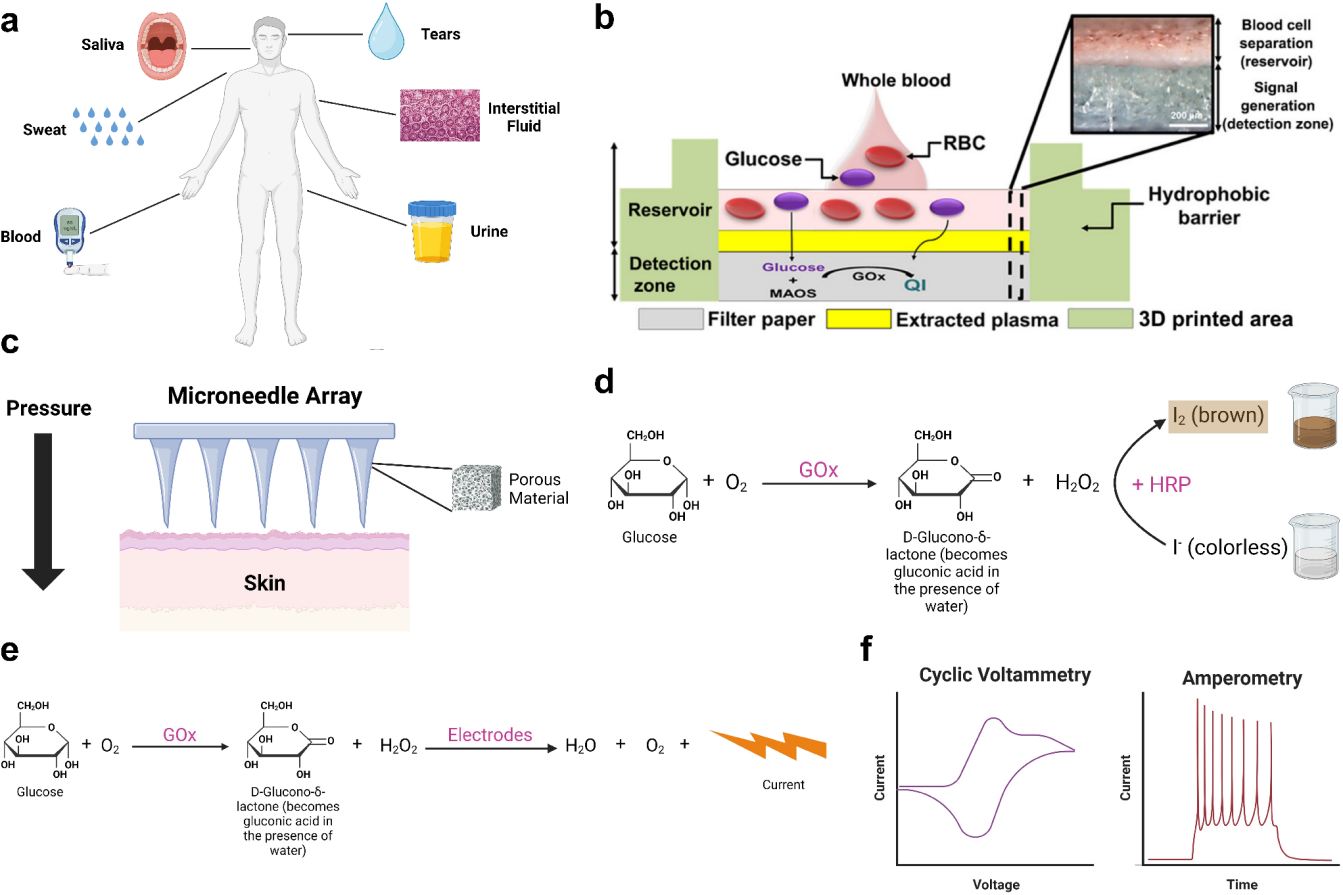
Example microfluidic devices and detection methods for glucose testing. **a** A schematic illustrating bodily fluids, including blood, interstitial fluid, saliva, sweat, tears, and urine, that have been explored for glucose detection. **b** A paper-based microfluidic blood glucose testing device with colorimetric glucose detection [64]. **c** Porous microneedle array-driven extraction of interstitial fluid for glucose testing (recreated from [74]). **d** An example chemical reaction in colorimetric glucose detection with iodide as the chromogenic agent, which is reduced to brown-colored molecular iodine in the presence of H_2_O_2_. Other chromogens such as a mixture of 4-aminoantipyrine (AAP) and 3,5-dichloro-2-hydroxybenzenesulfonic acid (DHBS) can be used in place of iodide [80]. **e** An example electrochemical assay for glucose detection, which generates a detectable current upon the reduction of H_2_O_2_. **f** Cyclic voltammetry and amperometry, two commonly used methods to characterize the electrical current generated by an electrochemical glucose detection system. (**a**, **d**, **e**, **f**) Created with Biorender. (**b**) Reproduced from Park C, Kim HR, Kim SK, Jeong IK, Pyun JC, Park S. Three-Dimensional Paper-Based Microfluidic Analytical Devices Integrated with a Plasma Separation Membrane for the Detection of Biomarkers in Whole Blood. ACS Appl Mater Interfaces. 2019;11:36428–36,434 [64]. Copyright permission from ACS Publications (CC License). (**c**) Recreated from Takeuchi K, Takama N, Kinoshita R, Okitsu T, Kim B. Flexible and porous microneedles of PDMS for continuous glucose monitoring. Biomed Microdevices. 2020;22:79 [74]

Glucose can be detected in microfluidic devices with various approaches, including colorimetric, electrochemical, fluorescence, chemiluminescence, and nanoparticle-based characterizations. Colorimetric and electrochemical measurements have been the two most commonly used methods (Fig. 1d).

Colorimetric assays are widely used because they are known for their simplicity, stability, user-friendliness, high throughput, low cost, and instrument-free nature [62, 63, 80, 82, 92]. For colorimetric glucose detection, glucose oxidase (GOx) is typically used to oxidize glucose into D-glucono-δ-lactone (or gluconic acid in the presence of water) and hydrogen peroxide (H_2_O_2_). Horseradish peroxidase (HRP) is typically used to convert an added chromogen into a colorful state in the presence of H_2_O_2_, or vice versa [63, 64, 80, 81].

Electrochemical approaches are also often used to detect glucose in microfluidic devices. Compared to colorimetric assays, electrochemical assays are typically less user-friendly due to more complicated instruments and procedures, but they are known for an even higher sensitivity and resolution [100]. Glucose-sensing microfluidic electrochemical assays typically involve a circuit, containing electrodes and a conductive medium, that can convert the glucose concentration into an electrical current. These electrodes are usually made of carbon-based materials, such as graphite [60, 65], biochar [66], carbon nanotubes [73], or graphene [72], doped with conductive materials such as metal nanoparticles or ions [66, 72, 73], or Prussian blue [60, 67]. Silver/silver chloride (Ag/AgCl) often serves as reference electrodes, and current is generated from reactions such as the aforementioned oxidation of glucose catalyzed by GOx [65, 83, 87, 90]. Amperometry, cyclic voltammetry, and linear sweep voltammetry are general techniques to correlate the current with the glucose concentration [65, 66, 68, 73].

### Bodily fluids for microfluidic glucose detection

A wide range of studies have been performed to detect glucose from various bodily fluids, including blood, interstitial fluid, saliva, sweat, tears, and urine, using microfluidic technologies (Fig. 1a). Most of these studies have clinically tested the efficacy of these custom-built, glucose-monitoring instruments. Therefore, we will mostly focus on the clinical results of these studies as well as the technologies they used for glucose detection from each bodily fluid.

#### Blood

As the gold standard for detecting and monitoring diabetes, blood glucose tests have been widely performed and incorporated into microfluidic devices. Traditional finger prick methods have been used to obtain blood from human subjects, and blood (or pre-processed plasma or serum) is loaded into various types of microfluidic devices (an example is shown in Fig. 1b, [64]). Numerous researchers have shown that microfluidic devices are capable of measuring blood glucose levels as accurately as or more accurately than traditional methods including colorimetry, high-performance liquid chromatography (HPLC), and commercially available blood glucose meters [60, 62, 64, 66–71]. Diabetes can also be quite accurately diagnosed using microfluidic-based glucose assays, along with microfluidic detection of other relevant chemicals such as cholesterol and triglycerides [64, 69].

#### Interstitial fluid

Although blood tests have been used as the clinical gold standard, they can be painful, stressful, and cause infections according to some users [3, 9, 11, 13–17]. Furthermore, subjecting fingertip tissue to chronic pricking can result in scarring and loss of finger sensation [101]. Therefore, instead of using sharp cannulas to draw blood, researchers have developed minimally invasive, microneedle-based systems for interstitial fluid extraction and glucose testing [72–79] (Fig. 1c). For example, Ribet et al. (2018) developed an integrated system consisting of a hollow silicon microneedle for drawing interstitial fluid and a microfluidic electrochemical sensing probe [76]. Takeuchi et al. (2019 and 2020) developed porous microneedle arrays using a salt leaching method that can be used to draw interstitial fluid for glucose testing [74, 75]. Although the detection of glucose from interstitial fluid is less invasive than blood glucose tests, it has an approximately 10-minute lag time attributed to the time taken for glucose to flow from the bloodstream into the interstitial fluid [76–79]. As long as there is sufficient modeling and correlation analysis, this lag time should be allowable, unless in an emergency [76].

#### Saliva

Because of the lag time and some invasiveness, using interstitial fluid for diabetes detection is still not ideal. Bodily fluids that can be obtained without any invasive penetration, such as saliva, urine, sweat, and tears, have also been used for glucose detection in diabetes monitoring. Thanks to the ease of obtaining saliva and its use as a well-studied diagnostic fluid, it has been most widely used of this group in the detection of glucose [102]. Numerous researchers have proposed microfluidic devices, most commonly paper-based devices, that readily detect glucose from saliva using colorimetric or electrochemical assays [66, 68, 80–86]. Salivary glucose concentration was found to be significantly higher in diabetic patients as compared to healthy subjects and can be used as an accurate indicator of hyperglycemia [80, 81, 85].

#### Sweat, tears, and urine

Similarly, sweat, tears, and urine have been used as alternative bodily fluids for non-invasive glucose detection, although less commonly than saliva. Sun et al. (2022), Bolat et al. (2022), and Xiao et al. (2019) successfully detected sweat glucose concentrations using custom-made microfluidic devices with electrochemical (Sun and Bolat) or colorimetric (Xiao) assays [83, 87, 88]. They found that glucose levels significantly increased upon consumption of a glucose-rich meal or solution, and Bolat et al. found that sweat glucose correlated very well with blood glucose, indicating that it is an accurate indicator of hyperglycemia. Allameh et al. (2022) and Agustini et al. (2017) measured tear glucose using µPADs with a distance-based colorimetric assay and µTEDs with an electrochemical assay, respectively [89, 90]. Agustini et al. indicated that tear glucose levels correlated very well with blood glucose levels [90]. Wei et al. (2021) suggested that similar to blood and saliva, urine glucose concentrations can be accurately measured using a hybrid microfluidic sensor [68]. Sechi et al. (2013) indicated that urine glucose was significantly increased in diabetic patients as compared to healthy subjects, which means that urine glucose can also be used as a metric for diabetes diagnosis [91]. However, most urine glucose studies are still in the preclinical stage with synthetic urine testing [84, 92, 93].

### Other methods of diabetes monitoring

Although glucose quantification is the gold standard for diagnosing and monitoring diabetes, it has obvious limitations. For instance, glucose concentrations are known to have diurnal variations, and factors such as diet, exercise, stress, illness, and insulin resistance all impact glucose levels in the blood and other body fluids [24, 94–96].

#### HbA1c quantification

**Fig. 2:**
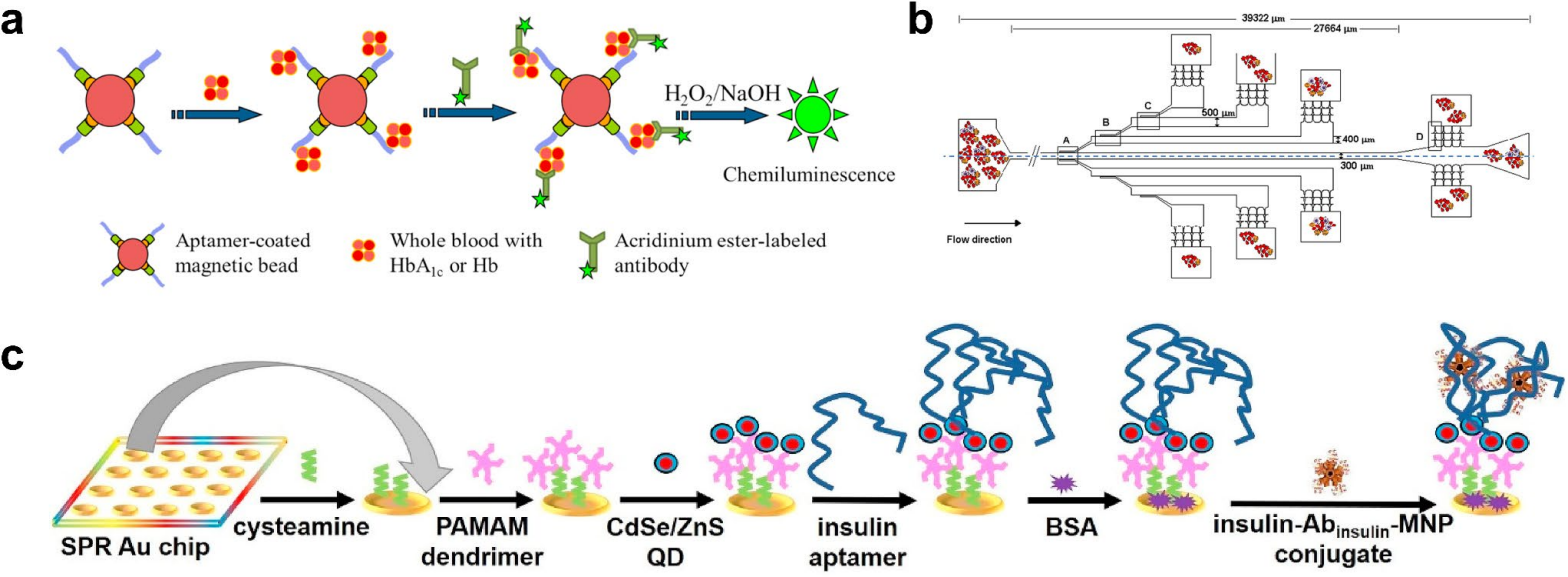
Microfluidic diagnosis of diabetes using methods other than glucose tests. **a** An aptamer-antibody assay used to quantify total hemoglobin (Hb) and glycated hemoglobin (HbA1c) [95]. **b** A single-inlet (I), multi-outlet (O1-O9) microfluidic device for red cell deformability measurement [27]. **c** A surface plasmon resonance sensor array for serum insulin detection using dendrimers and aptamers [98]. (**a**) Reproduced from Chang KW, Li J, Yang CH, Shiesh SC, Lee GB. An integrated microfluidic system for measurement of glycated hemoglobin levels by using an aptamer-antibody assay on magnetic beads. Biosens Bioelectron. 2015;68:397–403 [95]. Copyright permission from Elsevier. (**b**) Recreated with BioRender from Pinho D, Faustino V, Catarino SO, Pereira AI, Minas G, Pinho FT, Lima R. Label-free multi-step microfluidic device for mechanical characterization of blood cells: Diabetes type II. Micro and Nano Engineering 2022;16:100149 [27]. Copyright permission from Elsevier. (**c**) Reproduced from Singh V. Ultrasensitive quantum dot-coupled-surface plasmon microfluidic aptasensor array for serum insulin detection. Talanta. 2020;219:121314 [98]. Copyright permission from Elsevier

Compared to glucose tests, HbA1c tests have been suggested to be a more accurate indicator of diabetes [95, 103–105]. The most commonly used metric for HbA1c is the ratio between the concentration of HbA1c and total hemoglobin (Hb) [106–108]. The gold standard for HbA1c testing is high-performance liquid chromatography (HPLC), which is accurate but requires large-scale, costly equipment [95]. To minimize the scale and cost of HbA1c testing, Kuan et al. (2016) fabricated a polymethyl methacrylate (PMMA) microfluidic device integrated with dual complementary metal-oxide-semiconductor (CMOS) polysilicon nanowire sensors [94]. They measured total Hb and HbA1c from whole blood using a sodium lauryl sulfate hemoglobin assay and a miniaturized cation-exchange HPLC, respectively, in a time-efficient manner with a minimal amount (5 µL) of blood. Considering the peptide nature of Hb, some researchers performed immunoassays instead of HPLC to quantify HbA1c. For example, Chang et al. (2015) conducted an aptamer-antibody assay that attached Hb- and HbA1c-specific aptamers to magnetic beads loaded in a microfluidic chip (Fig. 2a) [95]. Chemiluminescence was induced upon the addition of anti-Hb or anti-HbA1c secondary antibodies labeled with acridinium ester and substrates including H_2_O_2_ and NaOH. Similarly, Wu et al. (2015) measured HbA1c with chemiluminescence in a microfluidic chip with a two-antibody assay [96].

#### Insulin quantification

Cohen et al. (2017) developed a real-time insulin quantification system to determine time varying demand for insulin due to the fluctuation of insulin resistance and pharmacokinetics, which cannot be addressed by glucose monitoring [24]. They detected insulin levels in serum through a custom-made microfluidic chip loaded with microspheres conjugated with streptavidin and biotinylated anti-insulin. Similarly, Furutani et al. (2018) developed a rapid enzyme-linked immunoassay (ELISA) with a six-layer disc-shaped microfluidic device to detect insulin and other glucose-regulating proteins including adiponectin and leptin [97]. Singh (2020) considered limitations of standard immunoassays, including the addition of toxic chemicals and lengthy procedures, and took a different approach. They developed a surface plasmon resonance-based insulin sensor array utilizing aptamers and quantum dots (Fig. 2c) [98].

#### Biomechanical testing

Besides biochemical markers, mechanical characteristics of cells can also be altered by diabetes. For instance, red blood cells (RBCs) are known to become stiffened and less deformable in diabetic patients, compared to those in healthy subjects [27–30]. Leveraging this phenomenon, Pinho et al. (2022) measured RBC deformability using a PDMS microfluidic device containing several carefully designed cross-flow filtration barriers (Fig. 2b) [27]. They found that RBC deformability was approximately 0.3 for T2D patients as compared to 0.5 for healthy subjects. This change can be used as a metric for clinical diagnosis of diabetes in the future. In addition, diabetes is also marked by impaired chemotaxis of neutrophils, which is caused by high concentrations of glucose and advanced glycation end products [31].

## Use of microfluidic devices in diabetes treatment

The treatment of diabetes is centered upon insulin delivery for all T1D patients and between 20% and 30% of T2D patients [8, 32]. Using microfluidic devices, insulin can be delivered transdermally (across the skin), orally, intraperitoneally, or through inhalation. Alternatively, small molecules such as metformin, as well as other peptides such as glucagon-like peptide 1 (GLP-1) and GLP-1 receptor agonists can be used to treat diabetes. Microfluidic technologies used to treat diabetes are summarized in Table 2.

**Table 2:** Summary of literature describing microfluidic technologies on the treatment of diabetes [46, 47, 109–153]

Drug delivered	Delivery approach	Delivery tool	References
Insulin	Transdermal	Hollow microneedle arrays	[109–126]
		Solid microneedle arrays for permeation of liquid insulin	[47, 115, 127–131]
		Porous microneedle arrays	[132–134]
		Iontophoresis-assisted microneedle arrays	[47, 128, 133]
		Piezoelectric micropumps	[118, 122, 123, 125, 135, 136]
		Membrane-driven micropumps	[120, 121, 124, 137]
		Microfluidic devices with cellular and/or enzymatic components	[119, 138–140]
	Oral	Micro- or nanocarriers produced by microfluidic devices	[141–143]
		Robotic microinjectors	[144]
	Intraperitoneal	Implantable microfluidic devices	[144–146]
	Inhaled	Nebulizer	[147]
Metformin	Transdermal	Smart sensor-integrated microneedle arrays	[148]
	Oral	Micro- or nanocarriers produced by microfluidic devices	[149–151]
GLP-1 and its receptor agonists	Transdermal	Dissolving microneedle arrays	[152]
	Oral	Micro- or nanocarriers produced by microfluidic devices	[153]

## Insulin delivery

Current insulin delivery techniques have obvious limitations including painful cannula insertion, interference with activity, and embarrassment [42–47]. To alleviate these problems, researchers have leveraged microdevices including microfluidic chips, microneedle arrays, and nanoparticle- or microcapsule-based drug carriers. Unlike microfluidic-based diagnosis and monitoring of diabetes, the use of microfluidics and related devices for insulin delivery is still largely in the preclinical stage. Experiments with laboratory animals and in vitro examinations have been mainly used to confirm the efficacy of these products, and human subjects have been used in very few studies [113, 133, 154]. Subcutaneous injection is the most common approach for microdevice-based insulin delivery, yet other researchers seek to deliver insulin with even less invasive approaches including oral administration and inhalation. In this section, we will review recently developed microfluidics and related devices on the aforementioned approaches of insulin delivery, as well as their efficacy in glucose control.

### Microfluidic devices for transdermal insulin delivery

Due to enzymatic digestion and impervious epithelia in the gastrointestinal (GI) tract, insulin is conventionally delivered through a subcutaneous rather than oral approach [41, 141, 142, 155, 156]. However, considering the non-adherence of patients due to painful cannula insertions [48–51, 54, 55], it is imperative to develop less invasive alternatives such as microneedle arrays. Various types of microneedle arrays have been developed for insulin delivery, and these microneedle arrays can be integrated with components such as microfluidic pumps, pancreatic islets or cells, and enzymes for active, controlled insulin release.

#### Insulin delivery using microneedle arrays

**Fig. 3:**
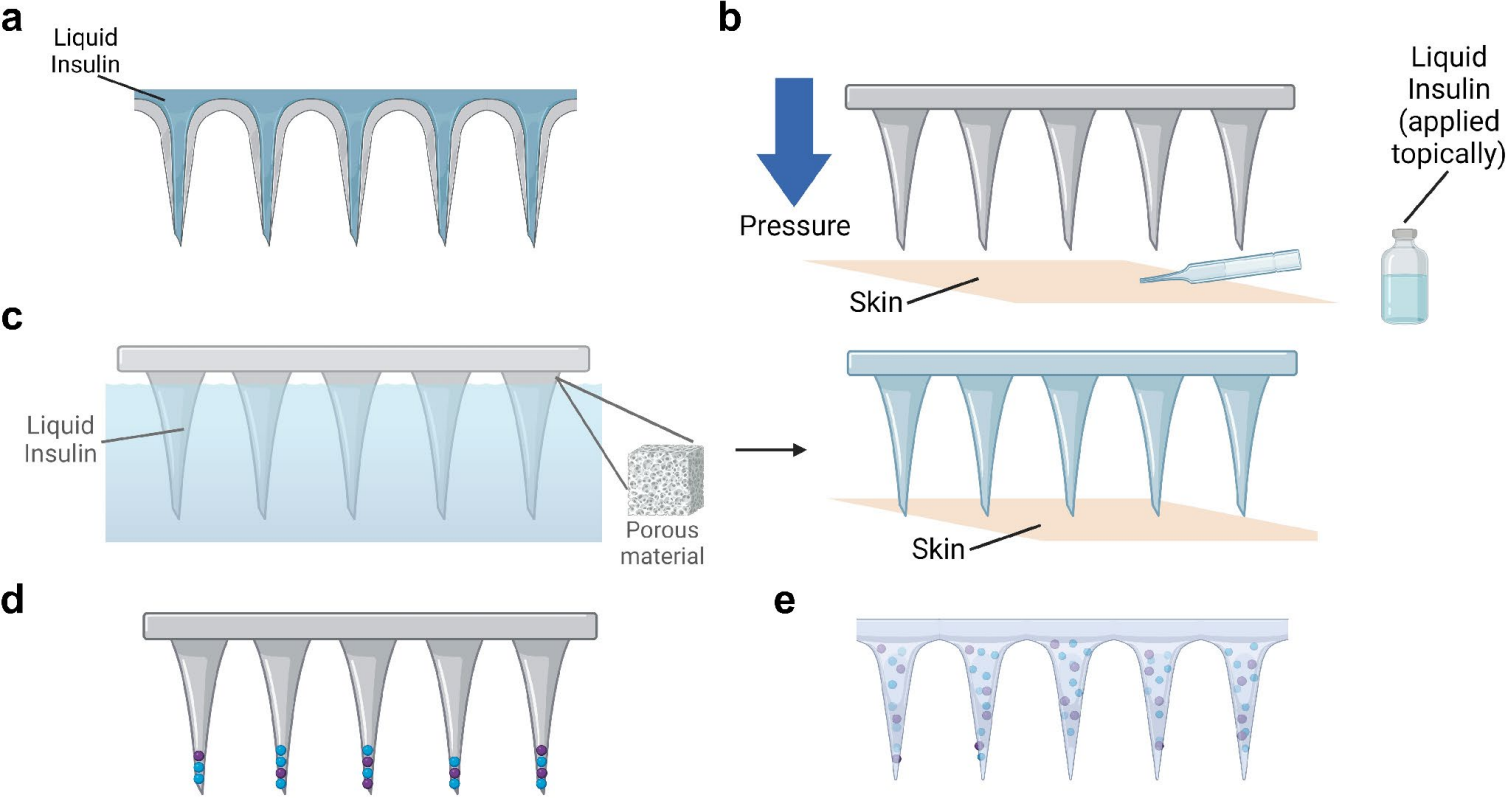
Different types of microneedle arrays for transdermal insulin delivery. **a** A hollow microneedle array used to inject liquid insulin across the skin. **b** A solid microneedle array used to pre-permeate the skin for more efficient topical insulin treatment. **c** A porous microneedle array for liquid insulin absorption and injection. **d** A solid microneedle array coated with insulin-containing polymer. **e** A hydrogel/dissolving microneedle array containing insulin and glucose-sensitive enzymes. (Blue particles: micro/nanoparticles containing insulin; maroon particles: micro/nanoparticles containing enzymes). Created with Biorender

**Fig. 4:**
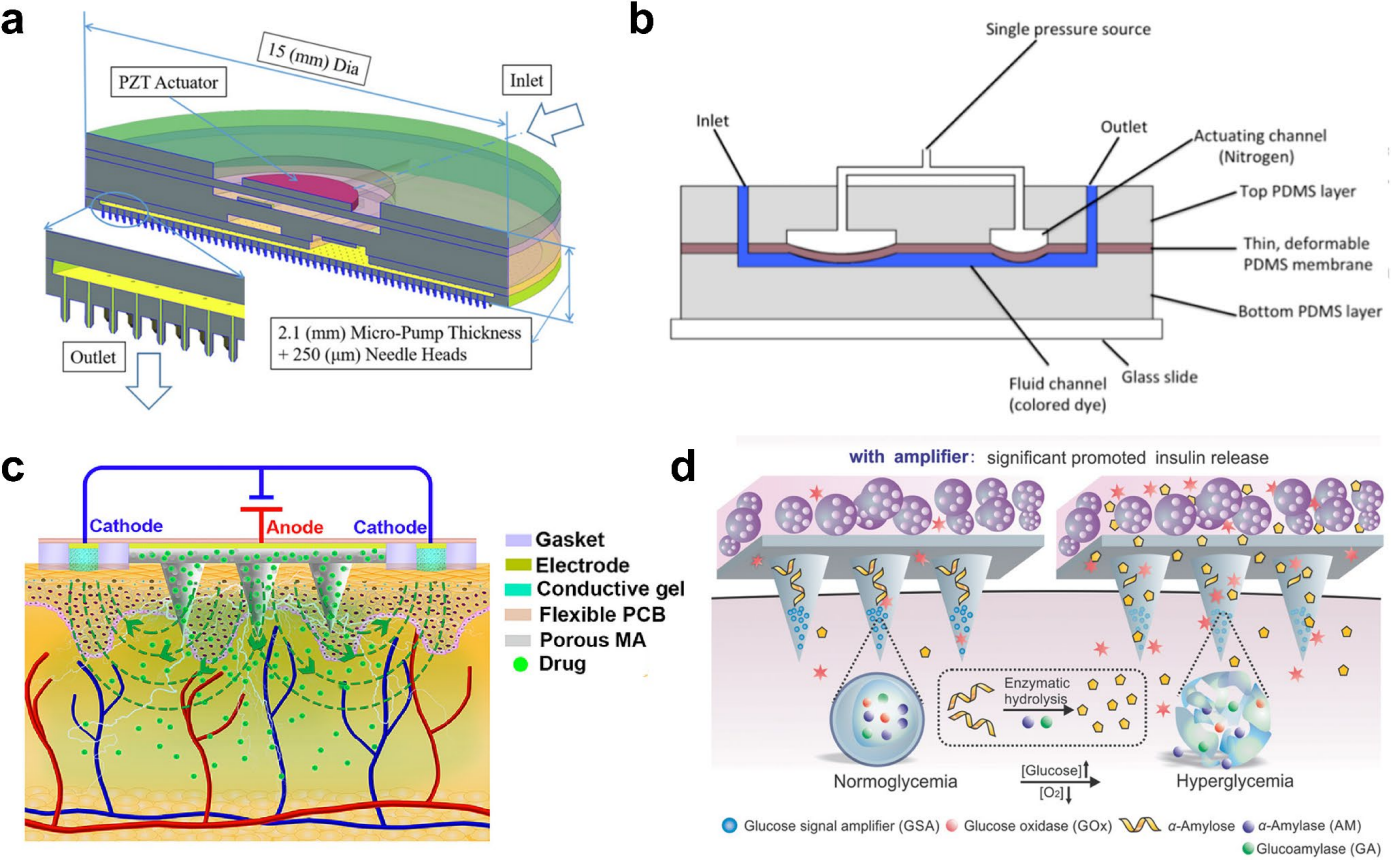
Technologies enabling active delivery of insulin across the skin compatible with a microneedle array-based system. **a**. A silicon-glass-PDMS-lead zirconate titanate (PZT) piezoelectric micropump integrated with a hollow microneedle array [123]. **b** An insect-mimetic, pulse-driven mechanical microfluidic pump from Chatterjee et al. [192, 193]. Zhang et al. (2022) later coupled the system with a 3D-printed hollow microneedle array [120]. **c** A hydrogel-based iontophoretic system that helps deliver insulin across the skin coupled with a porous microneedle array [133]. **d** A system that contains pancreatic islets that actively secrete insulin, as well as a dissolving microneedle array containing glucose signal amplifiers (GSA) including GOx, α-amylase (AM), and glucoamylase (GA) for glucose-sensitive insulin delivery [119]. (**a**) Reproduced from Meshkinfam F, Rizvi G. A MEMS-Based Drug Delivery Device With Integrated Microneedle Array—Design and Simulation. J Biomech Engi. 2021;143:081010 [123]. Copyright permission from American Society of Mechanical Engineers (ASME). (**b**) Reproduced from Chatterjee K. Analytical and Experimental Investigation of Insect Respiratory System Inspired Microfluidics: Virginia Tech; 2018 [192]. Copyright permission from Virginia Tech Libraries. (**c**) Reproduced from Li Y, Yang J, Zheng Y, Ye R, Liu B, Huang Y, Zhou W, Jiang L. Iontophoresis-driven porous microneedle array patch for active transdermal drug delivery. Acta Biomater. 2021;121:349–358 [133]. Copyright permission from Elsevier. (**d**) Reproduced from Ye Y, Yu J, Wang C, Nguyen NY, Walker GM, Buse JB, Gu Z. Microneedles Integrated with Pancreatic Cells and Synthetic Glucose-Signal Amplifiers for Smart Insulin Delivery. Adv Mater. 2016;28:3115–3121 [119]. Copyright permission from Elsevier

**Fig. 5:**
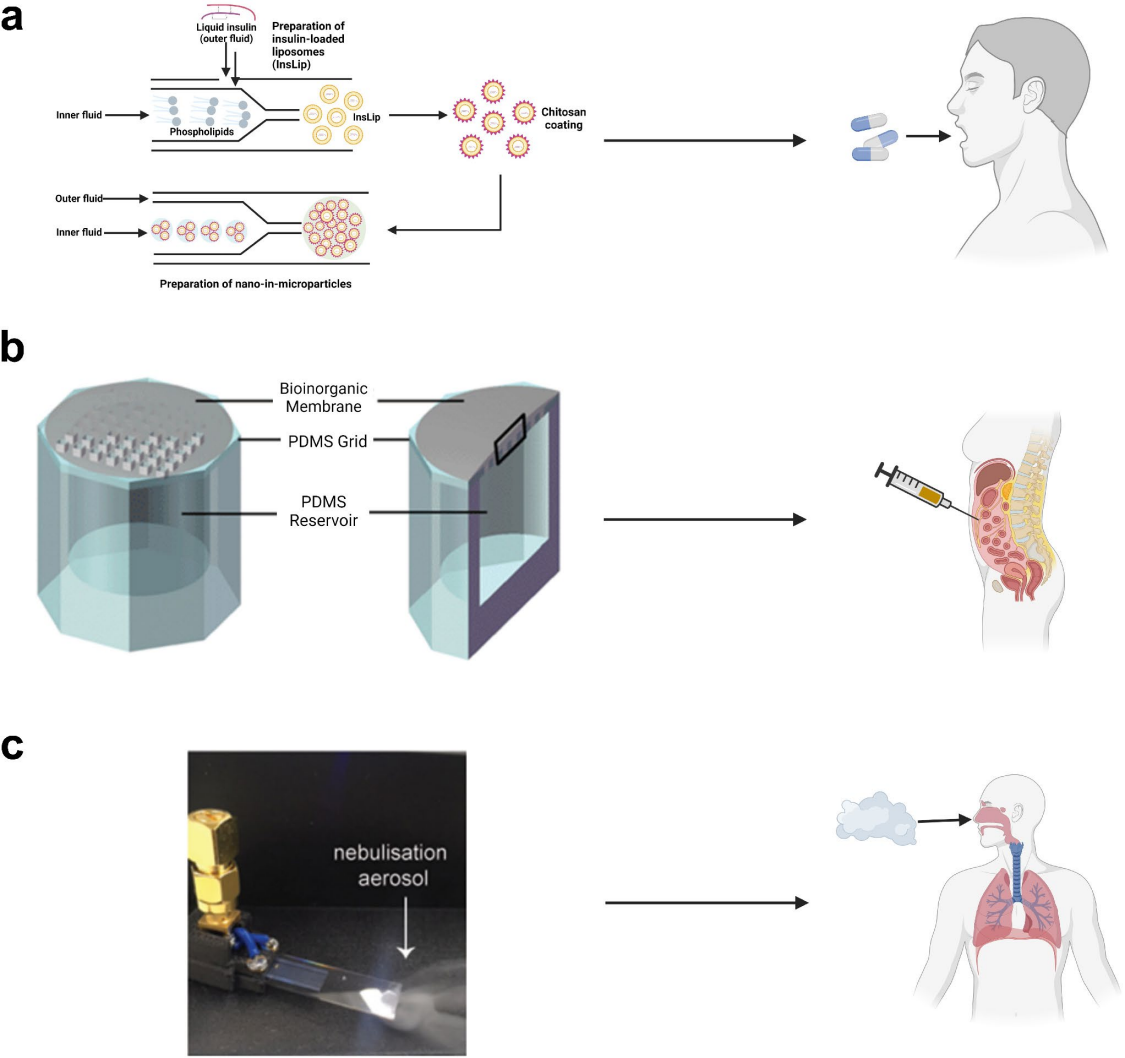
Oral administration, intraperitoneal injection, and inhalation of insulin enabled by microfluidic systems. **a** A microfluidic device used to produce microparticles containing chitosan-coated, insulin-encapsulated nanoparticles [141]. **b** A PDMS microfluidic insulin reservoir integrated with a bioinorganic gel membrane [146]. **c** A hybrid resonant acoustics (HYDRA) microfluidic nebulizer for insulin inhalation [147]. (**a**) Recreated using Biorender.com from Costa C, Liu Z, Martins JP, Correia A, Figueiredo P, Rahikkala A, Li W, Seitsonen J, Ruokolainen J, Hirvonen SP, Aguiar-Ricardo A, Corvo ML, Santos HA. All-in-one microfluidic assembly of insulin-loaded pH-responsive nano-in-microparticles for oral insulin delivery. Biomater Sci. 2020;8:3270-3277 [141]. Copyright permission from RSC Publications (CC License). (**b**) Reproduced from Chu MK, Chen J, Gordijo CR, Chiang S, Ivovic A, Koulajian K, Giacca A, Wu XY, Sun Y. In vitro and in vivo testing of glucose-responsive insulin-delivery microdevices in diabetic rats. Lab Chip. 2012;12:2533-9 [146]. Copyright permission from RSC Publications (CC License). Modified with Biorender.com. (**c**) Reproduced from Nguyen EP, Lee L, Rezk AR, Sabri YM, Bhargava SK, Yeo LY. Hybrid Surface and Bulk Resonant Acoustics for Concurrent Actuation and Sensing on a Single Microfluidic Device. Anal Chem. 2018 Apr 17;90(8):5335-5342 [147]. Copyright permission from ACS Publications (CC License). Modified with Biorender

Thus far, microneedle arrays have been the most commonly explored minimally invasive tool for transdermal insulin injection, considering their slim and lightweight nature and their ability to easily penetrate the dermis without injection site reactions [117]. Various insulin-delivering microneedle arrays have been designed, including (1) hollow microneedle arrays for direct insulin injection (Fig. 3a) [109–126], (2) solid microneedle arrays for insulin permeation (Fig. 3b) [47, 115, 127–131], (3) porous microneedle arrays soaked in insulin [132–134] (Fig. 3c), (4) insulin-coated microneedle arrays [46, 157–160] (Fig. 3d), and (5) hydrogel or dissolving microneedle arrays containing insulin [161–190] (Fig. 3e).

(I) **Hollow microneedle arrays**: Hollow microneedle arrays have been widely used for transdermal insulin delivery in research due to their ability to inject fluid into the skin without the need for additional components [113]. Mechanical tests proved that most of these microneedle arrays are mechanically durable (require a higher force to bend/fracture the needles than to penetrate the skin) [109–116], while transdermal tests confirmed that they can effectively release insulin into the skin [109, 115, 116]. To study the effect of insulin delivered by these microneedle arrays on blood glucose, rodents with diabetes induced by streptozotoxin (STZ) are usually used. Vinayakumar et al. (2016), Davis et al. (2005), and Li et al. (2022) all suggested that insulin delivered by hollow microneedle arrays was able to lower the blood glucose to a normoglycemic level within several hours [111, 112, 114]. Furthermore, several studies showed that hollow microneedle arrays were able to achieve blood glucose reductions on par with subcutaneous injections (as positive controls) [109, 111, 114]. Resnik et al. [113] conducted a clinical study by delivering U-100 and U-200 insulin to a non-diabetic human subject using silicon hollow microneedle arrays. They found that the insulin-delivering microneedle arrays caused an immediate but modest glucose reduction, as opposed to a gradual but substantial drop in blood glucose caused by subcutaneous injections [113]. Moreover, as compared to subcutaneous injections, hollow microneedle arrays do not cause persistent injuries such as bleeding and erythema [117].
(II) **Solid microneedle arrays for liquid insulin permeation**: Solid microneedle arrays are also promising tools for insulin delivery. They are typically used to pre-permeate the skin or create microchannels in the skin before insulin is applied topically [47, 127, 128, 130]. An early study by Chen et al. (2009) showed that solid stainless steel microneedle arrays induced microchannels in the skin, which enhanced the transdermal diffusion of insulin (encapsulated in nanovesicles) by approximately two orders of magnitude [128]. Similarly, Zhang et al. (2020) suggested that silicon nano-microneedles were able to enhance the diffusion of insulin across the skin by a factor of 2.5 [130]. Since solid microneedle arrays cannot act as vehicles for injection, other methods are often used in combination with these microneedle arrays to enhance the delivery of insulin across the skin. For instance, solid microneedle arrays are often coupled with electrodes to deliver insulin through iontophoresis, since electrical stimuli can drive charged particles such as insulin across the skin [47, 128]. Another commonly used method is to pre-encapsulate insulin into micro- or nanoparticles since these particles can cross the stratum corneum more easily than free insulin [115, 128]. Mechanical “press-and-release” is another method to enhance the delivery of insulin into the skin [127]. The efficacy of insulin-delivering solid microneedle arrays in lowering blood glucose levels is similar to that of hollow microneedle arrays. Yang et al. (2018), Yang et al. (2020), Zhang et al. (2020), and Chen et al. (2009) showed that insulin delivered by solid microneedle arrays was able to induce normoglycemia in diabetic rats within several hours [47, 127, 128, 130]. An additional attractive feature of this insulin delivery method is that, compared to hypodermic injections, solid microneedles do not result in sharp hypoglycemic shocks [47, 127].
(III) **Porous microneedle arrays**: Instead of creating microchannels before insulin delivery, porous microneedle arrays can absorb liquid insulin and release insulin into the skin [132, 133]. These microneedle arrays are fabricated with special methods such as salt leaching, solid-state sintering, or the addition of porogens or surfactants [74, 75, 132–134]. Li et al. (2017) found that a titanium-based porous microneedle array increased the permeation of calcein through rabbit skin by 27 times [132]. Similar to solid microneedle arrays, porous microneedle arrays can also be coupled with iontophoresis [133]. Using an iontophoresis-driven microneedle patch, Li et al. (2021) found that the blood glucose of STZ-induced diabetic rats could be reduced to a normoglycemic level within 3 h [133]. Additionally, insulin delivered by porous microneedle arrays did not cause a hypoglycemic shock, similar to other microneedle array delivery methods [133]. The microneedle arrays could be integrated with a glucose-sensitive gating, so that insulin would only be delivered in hypoglycemic, but not normoglycemic, conditions [134].
(IV) **Other types of microneedle arrays**: Besides the aforementioned microneedle array types, other types of microneedle arrays have been developed, including dissolving microneedle arrays, hydrogel-based microneedle arrays, and solid microneedle arrays with an insulin-containing polymer coating. Hydrogel-based and dissolving microneedle arrays are especially promising because they can achieve slow and controlled release of insulin without polymer deposition in the skin [191]. Insulin is typically incorporated in the bulk polymer of these microneedle arrays, and glucose-sensitive components (such as glucose-responsive nanovesicles) are also often added into the polymer to facilitate closed-loop insulin delivery. Similarly, solid microneedle arrays can be coated with insulin-containing polymers, which can also be released into the skin [46, 157–160]. Since this article primarily focuses on microfluidics, we will not discuss these all-solid microneedle arrays in further detail.

#### Other microfluidic devices for transdermal insulin delivery

Microneedle arrays are proven to be a promising technology for insulin delivery, but they rely on passive diffusion and do not facilitate active pumping of insulin. Therefore, scientists have developed other microfluidic components, including iontophoretic devices (Fig. 4c), microfluidic pumps (Fig. 4a,b), and cellular and enzymatic components, and integrated these components with microneedle arrays to enhance the delivery of insulin (Fig. 4d).

(I) **Iontophoresis-assisted microneedle arrays**: Iontophoresis drives charged particles (such as insulin) across the skin barrier using electricity [133]. Iontophoresis is typically facilitated by placing electrodes (cathode and anode) on the skin. Conductive media, such as conductive hydrogels or films, are used to complete the circuit so that charged particles like ions and insulin contained in the skin can move through the circuit (Fig. 4c) [47, 133]. Iontophoresis was used as a technique for transdermal insulin delivery as early as 1997 [194]. In this study, Haga et al. developed a series of iontophoretic devices with electroplated copper electrodes in an agar gel. Using diabetic mice, they found that the blood glucose levels could be reduced by 60% within 90 min [194]. Iontophoresis has been used in tandem with microneedle arrays for insulin delivery in later studies. In this case, microneedle arrays can act as one electrode, and the other electrode is typically composed of Ag/AgCl [47, 128, 133]. Chen et al. (2009) found that iontophoresis enhanced the transdermal permeation of insulin by factor between 3.3 and 5.3 [128]. Both Yang et al. (2020) and Li et al. (2021) found that iontophoresis was able to further reduce blood glucose in diabetic rats (but not so much as to cause hypoglycemic shock) when used with nanoparticle-encapsulated insulin whose delivery was enhanced by solid microneedle arrays. Also, iontophoresis was shown to decrease the time it takes for microneedle array-injected insulin to induce normoglycemia in diabetic rats [47, 133].
(II) **Microfluidic pumps**: Mechanical pumping mechanisms can also facilitate the active delivery of insulin into the skin. Thus far, the overwhelming majority of microfluidic pumps used to drive transdermal insulin delivery are piezoelectric pumps [118, 122, 123, 125, 135, 136]. This is due to the ability of piezoelectric pumps to achieve a very high accuracy because they can change the pumping pressure drastically in response to a small change in voltage [171]. Briefly, piezoelectric pumps work by applying a voltage at a frequency across a piezoelectric membrane, which causes the membrane to deform, driving a fluid flow [118]. Using piezoelectric pumps, the delivery rate can be precisely controlled by fine-tuning the actuating voltage and frequency [118, 122, 123]. This makes the pumps ideal for precisely controlled insulin delivery. Besides piezoelectric pumps, numerous other types of mechanical microfluidic pumps can drive insulin delivery. Huang et al. (2007) constructed a PDMS-based micropump containing microchannels and microvalves coupled with glucose and flow sensors. Their micropump was designed to deliver insulin based on measured glucose levels and driven by the peristaltic deflection of the PDMS membranes [137]. Similarly, Chatterjee et al. (2021), Zhang (2021), and Zhang et al. (2022) developed insect-mimetic microfluidic pumps driven by periodic contractions of a PDMS membrane enabled by compressed air or the radial pulse on the human wrist [120, 121, 193] (Fig. 4b). Mishra et al. (2019) integrated hollow microneedle arrays into a Nafion membrane micropump for insulin delivery [124]. Instead of being driven directly by mechanical compressions, this micropump was actuated by a laser doppler vibrometer, which deformed the membrane as a function of the actuating voltage and frequency [124].
(III) **Microfluidic devices with cellular and enzymatic components**: A very different type of microfluidic device used to facilitate insulin delivery is one integrated with cellular components, specifically pancreatic islets or β-cells. Instead of using commercially available insulin, researchers can leverage the ability of these cells to secrete insulin in a glucose-sensitive manner enabled by inherent feedback mechanisms in these cells. Tendulkar et al. (2011) developed a microfluidic device containing pancreatic islets immobilized in alginate microbeads. They found that the insulin secreted by the islets could increase from 0.165 ± 0.059 ng/10 islets/min in normoglycemic conditions to 0.422 ± 0.095 ng/10 islets/min in hyperglycemic conditions [138]. Similarly, Quintard et al. (2022) incorporated human islets into a two-layer, pneumatically driven microfluidic pump and found that there was a significant increase in insulin secretion when subjected to hyperglycemic stimulation [139]. Enzymatic components can also be incorporated into microfluidic devices or microneedle arrays to facilitate glucose-sensitive insulin delivery. Chen et al. (2011) constructed a glucose-sensitive microfluidic device by incorporating a membrane containing pH-sensitive nanoparticles encapsulating GOx and HRP, which resulted in insulin being released 2.4 times faster in hyperglycemia compared to normoglycemia [140]. Incorporating both pancreatic β-cells and an enzymatic glucose-sensing system in a microfluidic device, Ye et al. (2016) demonstrated that there was only a generous secretion of insulin by β-cells in hyperglycemic conditions (Fig. 4d) [119].

### Microfluidic devices involved in oral insulin delivery

Oral delivery of insulin has advantages over transdermal routes because it increases patient compliance and can result in a more complete regulation and better re-activation of insulin-dependent glucose metabolism in the liver [141, 155]. Oral insulin delivery is challenging, however, because it is difficult for insulin to pass through the epithelium and withstand enzymatic digestion in the GI tract and acidic conditions in the stomach [142, 195, 196]. Researchers have developed numerous approaches to overcome these limitations, the most common of which is the encapsulation of insulin within micro- or nanovesicles. Microfluidic devices are commonly used to produce these carriers. Costa et al. (2020) used microfluidic devices to produce microparticles containing chitosan-coated, insulin-encapsulating liposomes, which enhanced the permeation of insulin across the intestinal wall (Fig. 5a) [141]. A similar study conducted by Ma et al. (2023) suggested that microcapsules composed of a zwitterionic copolymer produced by a microfluidic device helped insulin both survive stomach-like acidic conditions and pass through the intestinal wall. In vivo tests suggested that this oral delivery method maintained normoglycemia better than subcutaneous injections in diabetic mice [142].

Other microfluidic devices used in service of oral insulin delivery include insulin-loaded robotic microinjectors produced by microfluidic devices intended for oral administration, as developed by Ghosh et al. (2022) [144]. These microinjectors were used to mechanically penetrate the intestinal wall with microneedle-like structures (“arms”) and therefore deliver insulin into the bloodstream. The efficacy of these microinjectors was tested with gelatin, stomach, and intestinal tissue, and it was found that the amount of insulin delivered across the tissues by these microinjectors was much higher compared to free insulin. Ghosh et al. also delivered the insulin microinjectors into diabetic rats intrarectally and found that the efficiency of insulin delivery was higher than previously developed devices for GI tract insulin delivery by an order of magnitude [144].

### Microfluidic devices involved in intraperitoneal insulin delivery

Although the administration route is relatively invasive, pre-clinical and clinical tests of intraperitoneal insulin delivery have shown that this route may have advantages compared to traditional insulin delivery methods, including more stable glucose levels and less time spent in hyperglycemia and hypoglycemia in diabetic patients [197, 198]. Microfluidic platforms have been developed for intraperitoneal insulin delivery. For instance, Luo et al. (2023) used an approach in which an electrically driven microfluidic device was constructed to produce alginate- and cellulose-based droplets containing insulin-releasing β-cells [145]. These droplets were implanted into the peritoneal cavity of diabetic mice. They found that the blood glucose levels were lowered to a normoglycemic state within 2 h and stayed normoglycemic for 21 days [145]. Chu et al. (2012) developed microfluidic devices consisting of primary amine-activated PDMS and bioinorganic gel membranes containing MnO_2_ nanoparticles and *N*-isopropylacrylamide (NIPAM): methacrylic acid (MAA) hydrogel nanoparticles (Fig. 5b). The devices were intraperitoneally implanted into diabetic rats and the rats were able to maintain normoglycemia for 7 days [146].

### Microfluidic devices for inhaled insulin delivery

Compared to other insulin delivery approaches, the development of microfluidic technologies for inhaled insulin is rare, possibly owing to previous finding that use of inhaled insulin is positively correlated with lung cancer [199, 200]. Nguyen et al. (2018) fabricated a hybrid resonant acoustics (HYDRA) microfluidic nebulizer that could evaporate aqueous insulin into an aerosol ready for inhalation (Fig. 5c). Briefly, this device was fabricated with lithium niobate sputtered with chromium and aluminum and patterned with interdigitated transducers. Liquid insulin was deposited into the device and nebulized using acoustic waves. The nebulizer generated aerosolized insulin droplets with a mass-median aerodynamic diameter of 2.5 μm with a geometric standard deviation of 0.2 μm, which was ideal for alveolar deposition. They also found that the chemical structure of insulin was not damaged, but studies on the control of blood glucose levels using this technology have yet to be performed [147].

## Delivery of other drugs through microfluidic systems for diabetes treatment

Besides insulin, other pharmaceuticals can also lower blood glucose levels in diabetic patients, especially for type 2 diabetics [148–153, 201]. These pharmaceuticals include metformin, GLP-1, and GLP-1 receptor agonists. One advantage of these treatments over insulin therapy is that they do not tend to cause a hypoglycemic shock [152, 202].

### Metformin

Metformin controls blood glucose in type 2 diabetics by increasing insulin sensitivity without stimulating insulin secretion [203, 204]. Compared to insulin, which can cause lipid accumulation in the body, metformin is not obesogenic and can cause weight loss, which is beneficial for T2D patients [205]. Due to these benefits of metformin, some researchers developed microfluidic systems for metformin delivery. Lee et al. (2016) constructed a multi-layer microfluidic device integrated with various sensors and a dissolving microneedle array for the glucose-sensitive transdermal delivery of metformin. Metformin delivered by their system significantly decreased the blood glucose level of diabetic mice to a normoglycemic state within 4 h [148]. Joshi et al. (2020) and Cesur et al. (2020 and 2021) produced metformin-encapsulated microparticles such as niosomes and microbubbles with microfluidic systems intended for oral delivery but only characterized the release profile of metformin in vitro [149–151]. While Lee et al. (2016) successfully developed their integrated device for clinical trials to examine its efficacy in monitoring the glucose levels of human subjects [148], most studies on metformin delivery using microfluidic systems have been preclinical [149–151, 201].

### GLP-1 and its receptor agonists

Unlike metformin, GLP-1 and its receptor agonists can stimulate glucose-dependent insulin secretion [153]. Araujo et al. (2016) fabricated a glass-based microfluidic system used to encapsulate GLP-1 into microcapsules made of poly(lactic-*co*-glycolic acid) (PLGA) functionalized with chitosan and a cell-penetrating peptide (CPP) for effective penetration through the GI tract walls for oral GLP-1 delivery. They found that the microcapsules lowered the glucose levels of Type 2 diabetic rats to a normoglycemic state within 2 h and maintained normoglycemia for 6 h [153]. Chen et al. (2017) developed a microneedle array-based transdermal delivery system for a GLP-1 receptor agonist, exendin 4(Ex-4). Due to the inclusion of GOx, the delivery of Ex-4 was glucose-sensitive. The delivery of Ex-4 maintained normoglycemia in diabetic mice for 5 days without causing a hypoglycemic shock [152]. Similar to other approaches to treat diabetes with microfluidic technologies, the use of these technologies to deliver GLP-1 and its agonists remains in the preclinical stage.

## Conclusions

In this paper, we reviewed technologies in use and in development for diagnosing, monitoring, and treating diabetes using microfluidic systems. The diagnostic and monitoring technologies surveyed perform glucose tests on various bodily fluids or carry out other biochemical or mechanical assays. Compared to traditional blood glucose testing with finger pricks and glucometers, microfluidic-based glucose monitoring is advantageous due to its lightweight nature, reduced sample size, and high throughput [59, 206]. Additionally, microfluidic glucose monitoring from non-blood bodily fluids including interstitial fluid, saliva, sweat, tears, and urine greatly reduces the invasiveness of glucose tests compared to traditional methods. Besides glucose, other markers of diabetes, including glycated hemoglobin, insulin, and cellular responses, have been detected using microfluidic devices. Many researchers have conducted clinical studies and confirmed that these testing schemes are efficacious in healthy and diabetic human subjects [24, 27, 60–69, 71, 76, 79–83, 85, 88, 90, 91, 94, 96–98].

The microfluidic systems reviewed here for the treatment of diabetes delivered insulin, other peptides, or metformin through transdermal or intraperitoneal injection, oral administration, and inhalation. We note that very few studies on advanced microfluidic technologies for the treatment of diabetes have entered the stage of investigating clinical efficacy in human subjects [113, 148, 154]. Most studies have focused on preclinical testing using diabetic rodents and in vitro tissue samples. Translating research involving microneedle array-based transdermal drug delivery is considered challenging because such injections involve many unknown parameters such as microneedle geometry, the force required to insert microneedle arrays into the skin, as well as issues of sterilization, immunogenicity, and flow rate accuracy, which remains a major challenge [207]. Current insulin pump technologies deliver insulin to diabetic patients at meticulously controlled rates [208, 209]. Most noninvasive insulin delivery technologies will need further development to characterize their flow rate accuracies. The oral delivery of insulin and other peptide drugs still lacks approval for clinical trials [196], possibly due to their low oral bioavailability, which is a result of acidic conditions, enzymatic digestion, and epithelial barriers in the GI tract [196, 197]. Microfluidic platforms for inhaled insulin are rare, likely due to established correlations between inhaled insulin and lung cancer [199, 200]. The delivery of metformin using microfluidic technologies, on the other hand, faces fewer barriers. Metformin has already been approved by the US Food and Drug Administration for oral consumption, so extensive clinical trials are not needed to further examine its safety [210, 211].

The emerging microfluidic technologies discussed here have the potential to greatly reduce the inconvenience and discomfort of diagnosing, monitoring, and treating diabetes. Further characterization and refinement of the technologies is needed, followed by clinical trials and other studies involving human subjects, which will ensure the safety and efficacy of these technologies for treating diabetes in human patients.

## Data Availability

Due to the nature of this article as a review, no datasets have been generated in the work towards the completion of this article.
